# Belowground induction by *Delia radicum* or phytohormones affect aboveground herbivore communities on field-grown broccoli

**DOI:** 10.3389/fpls.2013.00305

**Published:** 2013-08-21

**Authors:** S. P. Pierre, S. Dugravot, M. R. Hervé, H. M. Hassan, N. M. van Dam, A. M. Cortesero

**Affiliations:** ^1^Institute of Genetic, Environment and Plant Protection (Mixed Research Unit 1349), Rennes 1 UniversityRennes, France; ^2^European University of BrittanyBrittany, France; ^3^Economic Entomology, Kafrelsheikh UniversityKafr El-Sheikh, Egypt; ^4^Department of Ecogenomics, Institute for Water and Wetland Research, Radboud University NijmegenNijmegen, Netherlands

**Keywords:** plant-mediated above-belowground interactions, induced plant defence, *Brassica* spp, root herbivory, phytohormones

## Abstract

Induced plant defence in response to phytophagous insects is a well described phenomenon. However, so far little is known about the effect of induced plant responses on subsequently colonizing herbivores in the field. Broccoli plants were induced in the belowground compartment using (i) infestation by the root-herbivore *Delia radicum*, (ii) root application of jasmonic acid (JA) or (iii) root application of salicylic acid (SA). The abundance of *D. radicum* and six aboveground herbivores displaying contrasting levels of host specialization were surveyed for 5 weeks. Our study showed that the response of herbivores was found to differ from one another, depending on the herbivore species, its degree of specialization and the root treatment. The abundance of the root herbivore *D. radicum* and particularly the number of emerging adults was decreased by both phytohormone treatments, while the number of *D. radicum* eggs was increased on conspecific infested plants. The root infestation exhibited moderate effects on the aboveground community. The abundance of the aphid *Brevicoryne brassicae* was strongly increased on *D. radicum* infested plants, but the other species were not impacted. Root hormone applications exhibited a strong effect on the abundance of specialist foliar herbivores. A higher number of *B. brassicae* and *Pieris brassicae* and a lower number of *Plutella xylostella* were found on JA treated plants. On SA treated plants we observed a decrease of the abundance of *B. brassicae*, *Pi. rapae*, and *P. xylostella*. Surprisingly, generalist species, *Mamestra brassicae* and *Myzus persicae* were not affected by root induction treatments. Finally, root treatments had no significant effect on either glucosinolate (GLS) profiles of the heads or on plant quality parameters. These results are discussed from the perspective of below- aboveground interactions and adaptations of phytophagous insects to induced plant responses according to their trophic specialization level.

## Introduction

Induced plant resistance against herbivores is a widespread phenomenon, reported for over one hundred plant species (Karban and Baldwin, 1997). Induced responses include physical and chemical alterations such as modification in leaf toughness, trichome density, as well as changes in the concentration of allelochemicals and nutritional compounds (Karban and Baldwin, 1997). In the field, such plant responses induced early in the season may affect subsequently colonizing herbivores (Shiojiri et al., 2002; Viswanathan et al., 2005; Poelman et al., 2008, 2010). The effect of induced plant responses may have contrasting effects on host-plant acceptance by herbivores belonging to different feeding guilds or having different levels of host specializations (Inbar et al., 1999; Agrawal, 2000; Denno et al., 2000). In particular, it has been found that generalist herbivores tend to avoid induced plants while specialists prefer to colonize such plants (Martinsen et al., 1998; Kaplan and Denno, 2007; Long et al., 2007; Poelman et al., 2008, 2010). This dissimilarity could result in important modifications of herbivore communities associated with individual plants depending on their induction status (van Zandt and Agrawal, 2004; Poelman et al., 2010). So far, studies on the influence of induced responses on herbivore community structure have been focused on the aboveground compartment. However, there is a growing awareness that plants and insects are part of complex and connected multitrophic aboveground and belowground communities (van Dam and Heil, 2011). Although aboveground and belowground responses are sometimes independent (Bezemer et al., 2003, 2004), they may also interact with each other. Indeed, several studies reported that root herbivory—or hormone applications as a substitute for root feeding—not only causes a local (root) but also a systemic (foliar) increase in levels of defence compounds [reviewed by Bezemer and van Dam (2005); Erb et al. (2008); van Dam and Heil (2011)]. Jasmonic acid (JA) is known to be a central signaling compound in plant defence induction against chewing insects (Howe and Jander, 2008) that is upregulated in the roots after belowground attack (Erb et al., 2009). Nevertheless its relative importance as a defence signal belowground is still unclear. Salicylic acid (SA) is known to be involved in responses to sap-sucking insects and pathogens, and can act as JA antagonist (Becker and Spoel, 2006). Topical applications of both hormones have been used successfully to mimic herbivore and pathogen induced response in plants (e.g., Moore et al., 2003). Although root and shoot chewers may elicit similar induction pathways, root induction can trigger different responses in the leaves when compared to leaf attack (Masters et al., 1993; van Dam et al., 2004).

Root-induced changes in plant defence can have major impacts on aboveground organisms feeding on the plant. In some cases, such plant induced responses result in lower performance of leaf herbivores (Bezemer et al., 2003; Soler et al., 2005; van Dam et al., 2005). In other cases, root herbivores also induce a stress response in the host plant similar to drought stress, leading to the reallocation of photoassimilates in shoots and, consequently, affecting positively or negatively the performance of foliar feeders (Masters et al., 1993; Huberty and Denno, 2004; Erb et al., 2011a). Therefore it is hard to predict the effects of root induced response on foliar herbivores without experimental assessments. A few authors have investigated whether aboveground induced plant responses to herbivory or phytohormone applications are costly by quantifying plant growth, survival and reproduction (e.g., Thaler, 1999; Agrawal, 2000; Redman et al., 2001; van Dam and Baldwin, 2001). However, to our knowledge thus far it has not been examined if root induction by insect herbivory or phytohormone applications affects the herbivore community associated with a crop and if such induction has any influence on the yield and nutritional quality of the harvested products.

*Delia radicum* L., the cabbage root fly, is a major pest of brassicaceous crops such as broccoli, Brussels sprouts, cabbages and kales. Females aggregatively lay their eggs near plant stems and the larvae crawl down to feed on the roots before pupating in the soil surrounding the roots. Plant mortality caused by larval damage may lead to significant reductions in yield, flowering, and seed production as well as in leaf, stem and root biomass (de Jong and Städler, 1999). This root herbivore is also a well-studied model system for plant-mediated above-belowground interactions. Experiments using cultivated and wild *Brassica* species showed that root feeding by *D. radicum* and its close relative *D. floralis*, induce notable changes in the levels of primary and secondary non-volatile compounds in the plant (Birch et al., 1992; Hopkins et al., 1998, 1999; Soler et al., 2005; van Dam et al., 2005; van Dam and Raaijmakers, 2006; Pierre et al., 2012) as well as changes in its volatile blend (Neveu et al., 2002; Soler et al., 2007; Pierre et al., 2011). In particular, *D. radicum* feeding on roots was shown to induce both a local (in roots) and a systemic (in shoots) increase of indole and aliphatic glucosinolates (GLS) levels in wild *Brassica* species (van Dam and Raaijmakers, 2006). This systemic induced response to *D. radicum* feeding could contribute at least partly to reduced performance of the leaf feeder specialist *Pieris brassicae* on feral *B. nigra* plants (Soler et al., 2005).

Here, we present a field experiment on broccoli (*Brassica olearacea* subps. *italica*) in which we specifically address the following questions: (i) does root herbivory, JA or SA root application affect plant colonization by subsequent herbivorous insects attacking the leaves or the roots of this plant? (ii) do herbivores of different feeding guilds and levels of host specialization differ in their response to root-induced plants? (iii) do the root induction treatments affect yield and quality of the crop?

## Materials and methods

### Plants

Cultivated broccoli plants, *Brassica oleracea* subsp. *italica* (var. Monaco) were used for the experiment. Seeds were sown individually in peat soil cylinders and grown during 6 weeks in a plastic tunnel (Technosem, Cleder, Brittany, France). The experimental site was located in the “Domaine experimental de la Motte” (INRA center, Le Rheu, Brittany, France). Before plantation, the soil was fertilized (100 N units) and treated with an herbicide (Butisan® S at a dose of 2L/Ha). Four days later (28th of April, 2009), the broccoli seedlings were transplanted into the soil at a density of 2.7 plants/m^2^. We used an anti-insect netting (Multiclima®, “Genetic et Distribution,” France) to prevent the plants from natural infestation before the beginning of the experimental survey. This net is transparent and made of U.V. treated *high-density polyethylene* (HDPE, 38 g/m^2^, 16% shade, with an average of 2°C difference from the ambient temperature). It is successfully used in various vegetable crops in Brittany as an alternative for pesticide treatments (Loïc Gueguen, pers. communication). Here the net was removed at a date (11th of June, 2009) corresponding to the beginning of the second *D. radicum* egg laying period and to the increased presence of various common *Brassica* pests such as *Plutella xylostella*, *Pieris brassicae*, and *Brevicoryne brassicae* (Bulletin de Santé du Végétal, DRAF Brittany website). At that time, the broccoli plants had 12–13 fully expanded leaves.

### Insects

For the plant infestation, we used *D. radicum* larvae originating from our rearing. The populations were originally established in the laboratory from flies collected in the field at St. Méloir des Ondes (Brittany, France) during the summer of 1994. The brood was reared as described in Neveu et al. (1996) and supplemented yearly with new field-captured individuals coming from the same area.

### Field experimental set up

A randomized complete block design was established at the same time as the anti-insect net was removed from the broccoli crop (11th of June 2009). Three blocks were constituted, each one included five randomized plots corresponding to the five different treatments tested. A plot consisted of 20 plants (five rows of four plants each). The plots were isolated from each other by a row of untreated broccoli plants. Induction treatments were applied 3 h after net removal. Infestation with *D. radicum* was carried out by placing five larvae (second and third instars) with a brush onto the soil surface immediately adjacent to the stem of each plant. This load of infestation was previously shown to trigger an alteration of the chemical profile of the plant (Pierre et al., 2011, 2012). Control plants for this induction treatment were plants that were not infested (C). Root treatments with JA and SA were made using the same procedure and the same phytohormone doses (i.e., 500 μg and 25 mg per plant, corresponding to 0.23 and 18 mM respectively) as described by van Dam et al. (2004) and for which induction of GLS was successfully demonstrated under controlled conditions (Pierre et al., 2012). To optimize induction in our field conditions, we repeated the phytohormone treatments two times during the experiment, the 22nd of June and the 6th of July. Control plants for such phytohormone inductions (AC) were treated with an acid solution (pH = 3.1 between pH of JA solution and SA solution) according to the same method as described by van Dam et al. (2004).

### Effect of induction on herbivore community

During the season, we surveyed six herbivorous species on the plants (Table 1). Other herbivores that were observed, namely flea beetles, whiteflies and thrips, were excluded from the survey as they could not be accurately counted without damaging the plants.

**Table 1:** **Herbivore species surveyed on *Brassica oleracea* subsp. *italica* (var. Monaco) experimental plots, and their degrees of host specialization**.

**Species**	**Order**	**Family**	**Feeding type**	**Host specificity**
*Delia radicum*	Diptera	Anthomyiidae	Root chewer	Specialist
*Pieris brassicae*	Lepidoptera	Pieridae	Leaf chewer	Specialist
*Pieris rapae*	Lepidoptera	Pieridae	Leaf chewer	Specialist
*Plutella xylostella*	Lepidoptera	Yponomeutidae	Leaf chewer	Specialist
*Mamestra brassicae*	Lepidoptera	Noctuidae	Leaf chewer	Generalist
*Brevicoryne brassicae*	Hemiptera	Aphididae	Phloem feeder	Specialist
*Myzus persicae*	Hemiptera	Aphididae	Phloem feeder	Generalist

From June 25th until July 15th, the four central plants of each plot were surveyed weekly by investigating both sides of all their leaves for the presence of herbivorous insects. In addition, egg laying by *D. radicum* was monitored weekly on two plants of each plot from June 18th until July 15th. For this purpose, felt traps were positioned around the stems of the plants, where flies deposit their eggs (Bligaard et al., 1999). These traps are used by farmers as an indicator of pest prevalence. The traps were collected and eggs found inside were counted and removed at each survey. Empty traps were then replaced on the same plants. In addition, at the harvest (described below), we dug out the entire roots and soil in a radius of 15 cm around the main root and at a depth of 20 cm, for three to six harvested plants per treatment group. The plants chosen were different from the ones used for monitoring *D. radicum* oviposition. These “root-soil” samples were put in separate plastic bags and brought back to the lab. Each sample was individually potted and covered with a micro-aerated plastic bag to control and survey *Delia* spp adult emergence. Based on identification of a representative sample of one hundred individuals under a binocular microscope, *D. radicum* individuals were found to be highly predominant, i.e., representing 99% of total *Delia* spp individuals, compared to the other species such as *D. antiqua* and *D. platura* which can also feed on Brassica plant roots and pupate in the soil. For this reason, all emerged adults were considered as belonging to *Delia radicum* species.

### Effects of induction on harvest yield and quality

The four central broccoli plants that were surveyed for herbivores and six other plants randomly selected from each plot were harvested on June 16th and 17th, which resulted into 10 plants ^*^ 3 blocks = 30 plants per treatment group. The height of each plant was measured before cutting it with clippers at 10–15 cm over the root-shoot interface. One hour later, each entire plant was weighed (balance Mettler PM 3000) with ±0.1 g accuracy. A general quality grade was assigned to each plant that was estimated as an average grade between commercial specifications for head quality (CEE-ONU FFV-48 standards reviewed) and visual aspect of the leaves. The classes were: Class1: leaves and head are green and without any apparent herbivore damage; head is firm, tight and deep green; Class 2: at least 90% of leaves and head are green and without apparent herbivore damage; at least 90% of head is firm, tight and deep green; Class 3: any other plant that does not match Classes 1 or 2.

The heads (cut with clippers to get 16–18 cm high heads, stem included) were weighed to determine their fresh biomass. Meanwhile, samples from 10 to 16 heads per treatment group were placed in separate paper bags to be stored at −20°C until they were freeze-dried to constant weight. A sub-sample of 90–110 mg was taken from each dried head sample and put individually in Eppendorf tubes for GLS profile analysis, by following similar procedures as described by van Dam and Raaijmakers (2006).

### Statistics

All statistical analyses were carried out with R software (version 2.14.0; R Core Team, 2011). Effect of treatment (*D. radicum* infestation or phytohormone application) on herbivore abundance was assessed using a Wald chi square test on a Generalized Linear Mixed Model, with a Poisson distribution (function “glmer” in package “lme4”; Bates et al., 2011). Data considered were the number of individuals/eggs per plant. Since the two control treatments (AC and C) were conducted differently, two models were built: one to test for an effect of phytohormone application, a second one to test for an effect of *D. radicum* infestation. In both models, treatment was specified as a fixed factor, date was specified as a random factor and block was specified as a random factor nested into date. The same procedure was used to test for an effect of treatment on abundance of *D. radicum* adults, without the date factor. When needed, pairwise comparisons were computed by using an analysis of contrasts [function “esticon” in package “doBy” (Højsgaard et al., 2011)] with a Benjamini-and-Hochberg correction.

Effect of treatment on plant quality (height, total weight, head weight and visual quality) was assessed by using a Wald chi square test on a Linear Mixed Model (function “lmer” in package “lme4”). Treatment was considered as a fixed factor and block as a random factor. When needed, pairwise comparisons were computed by an analysis of contrasts with a Benjamini-and-Hochberg correction.

Effect of treatment on GLS profile was assessed by using an Analysis Of Variance (ANOVA). Different ANOVAs were conducted to test for an effect on (i) total GLS, (ii) GLS classes (aliphatic and indole), and (iii) individual GLS content.

## Results

### *D. radicum* abundance

#### Effect of phytohormone treatment

**Figure 1:**
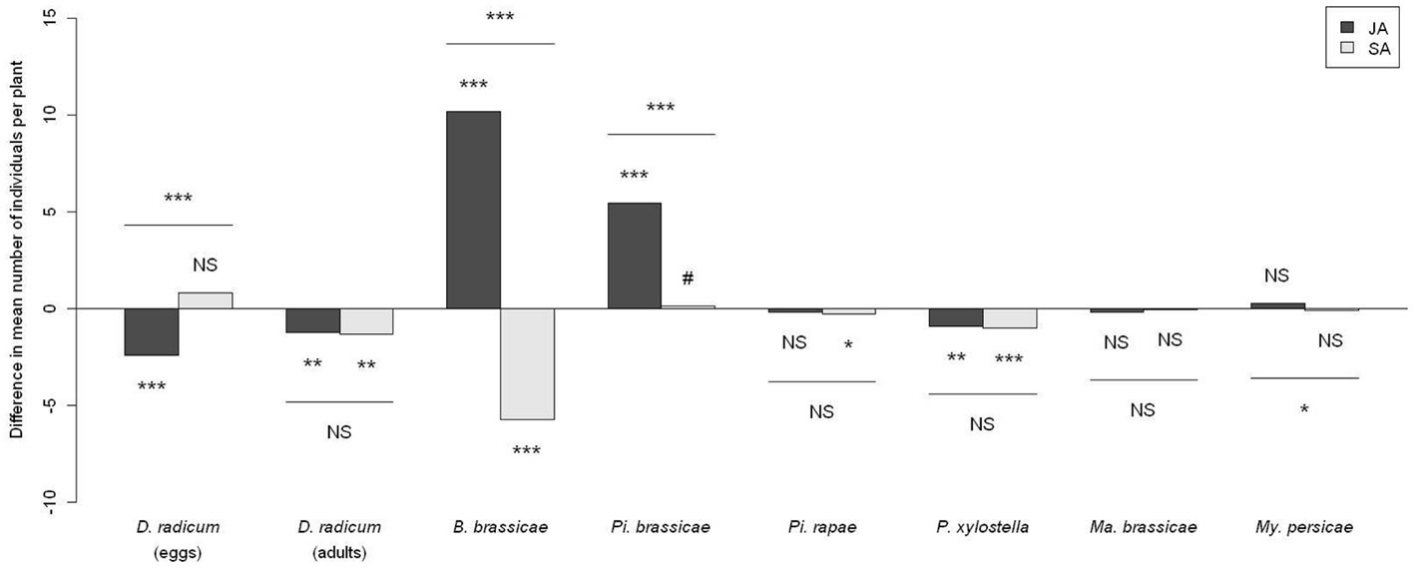
**Difference in herbivore abundance between phytohormone-treated plants and control plants.** Significancy of differences computed by an analysis of contrasts following a Wald test on a GLMM. JA, plants treated with jasmonic acid; SA, plants treated with salicylic acid. NS *P* > 0.10, ^#^ *P* < 0.10, ^*^P < 0.05, ^**^P < 0.01, ^***^P < 0.001.

Both egg abundance and adult emergence of the specialist root herbivore *D. radicum* were altered by phytohormone treatments (Figure 1). The number of eggs laid by *D. radicum* (*p* < 0.0001) was decreased on JA-treated plants but not on SA-treated plants (*p* = 0.25). Significantly lower numbers of adults emerged from JA (*p* = 0.0094) and SA treated plants (*p* = 0.0094) than from untreated plants.

#### Effect of *D. radicum* infestation

**Figure 2:**
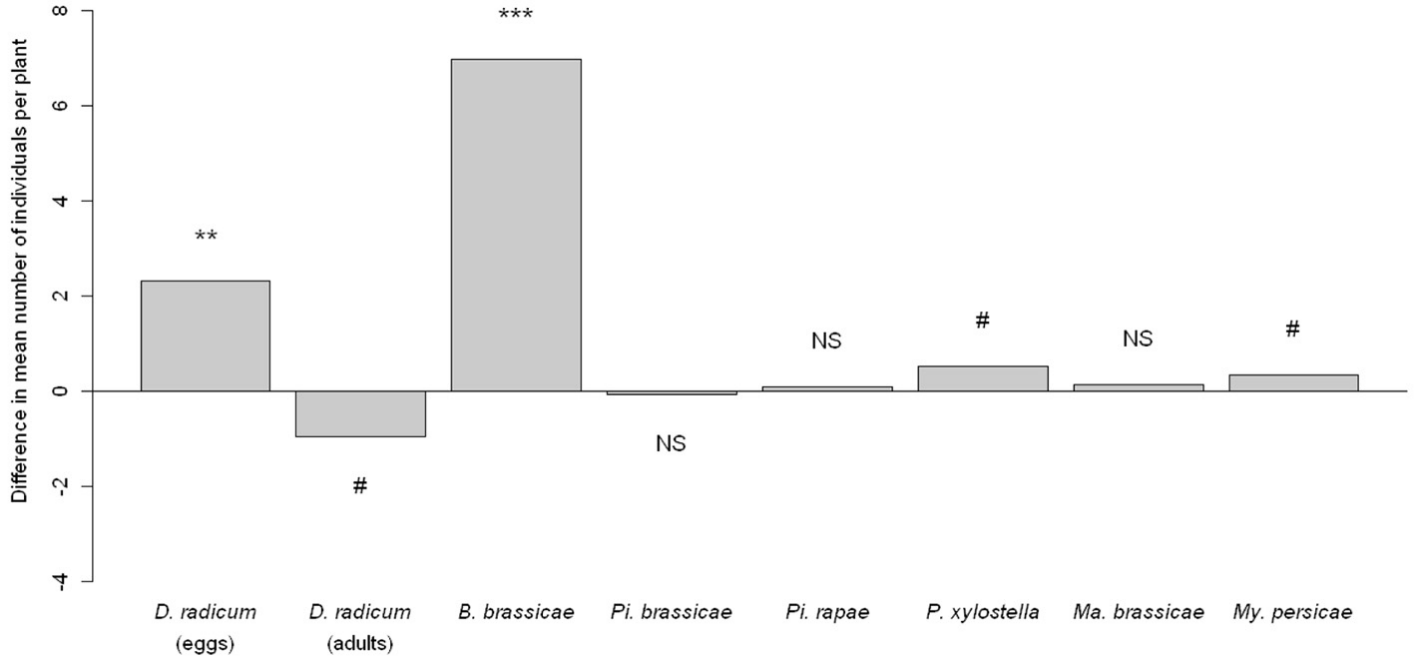
**Difference in herbivore abundance between *D. radicum*-infested plants and control plants.** Significancy of differences computed by a Wald test on a GLMM. NS *P* > 0.10, ^#^*P* < 0.10, ^**^P < 0.01, ^***^P < 0.001.

In contrast to the treatments with phytohormones, *D. radicum* infestation was clearly associated with higher numbers of conspecific *D. radicum* eggs (*p* = 0.0005) (Figure 2). However, the number of adults emerging from these previously infested plants was not different from control plants (*p* = 0.061).

### Foliar herbivore abundance

#### Effect of phytohormone treatment

Herbivore community development was globally affected by phytohormone treatments, however, the magnitude of the effect varied for some species and according to the phytohormone tested (Figure 1).

JA treated plants harbored more specialist *Pi. brassicae* larvae (*p* < 0.0001) and showed a higher abundance of the specialist *B. brassicae* (*p* < 0.0001). Conversely, JA application was associated with a lower abundance of the specialist *P. xylostella* (*p* = 0.001). This treatment however, did not affect the abundance of the other specialist *Pi. rapae* and the two generalists *My. persicae* and *Ma. brassicae*.

Conversely to JA, SA treated plants harbored fewer individuals of the specialist *B. brassicae* (*p* < 0.0001) but also fewer *Pi. rapae* (*p* = 0.019) and *P. xylostella* larvae (*p* = 0.0058). This treatment did not strongly affect the number of *Pi. brassicae* larvae (*p* = 0.0975). As for JA, SA applications had no influence on the abundance of the generalists *Ma. brassicae* and *My. persicae*.

#### Effect of *D. radicum* infestation

*Delia radicum* induced plants harbored more specialist *B. brassicae* individuals (*p* < 0.0001), more specialist *P. xylostella* larvae although the test was just not significant (*p* = 0.0592, Figure 2) but the abundance of all the other herbivores was not significantly affected by *D. radicum* infestation.

### Effect of treatments on harvest yield, quality and GLS head profiles

JA treated plants had reduced heights compared to control plants (34.5 ± 0.9 cm and 37.7 ± 0.7 cm, respectively; *df* = 85, *t* = 2.968, *p* = 0.004). By contrast, neither SA application nor *D. radicum* infest ation modified plant height compared to controls (36.6 ± 0.7 cm, 37.1 ± 0.7 cm and 36.7 ± 0.6 cm, respectively) (Table 2).

**Table 2:** **Effect of phytohormone treatments or *D. radicum* infestation on different parameters of plant quality (Wald tests on GLMMs)**.

**Variable**	**Phytohormone treatment**						***D. radicum* infestation**				
	**χ^2^**	***df***	***P-value***	**Control**	**JA**	**SA**	**χ^2^**	***df***	***P-value***	**Control**	**Infested**
Height (cm)	7.67	2	**0.0246**	37.70 (0.74) A	34.65 (0.95) B	36.60 (0.69) A,B	0.19	1	0.6641	36.68 (0.63)	37.10 (0.73)
Plant weight (g)	4.13	2	0.1268	2131.6 (62.5)	2082.8 (81.3)	1936.6 (69.2)	0.57	1	0.4497	2037.23 (69.87)	2109.33 (66.16)
Head weight (g)	2.41	2	0.2994	873.5 (48.3)	864.3 (57.2)	774.5 (46.0)	0.35	1	0.5518	826.48 (43.71)	863.67 (45.33)
Visual quality	0.68	2	0.7130	1.97 (0.10)	1.87 (0.11)	2.00 (0.14)	4.96	1	**0.0259**	2.13 (0.12)	1.73 (0.14)

Values presented are means (±SE). For phytohormones when the global effect is significant, different letters indicate statistically different means at P < 0.05. JA, plants treated with jasmonic acid; SA, plants treated with salicylic acid.

Bold value indicates *P* < *0.05*.

Phytohormone treatments did not change plant quality when considering visual aspects of their leaves and heads, whereas *D. radicum* infested plants showed lower quality than control plants (average quality grades were 1.7 ± 0.1 and 2.1 ± 0.1, respectively; *z* = −2.16, *p* = 0.0259). Neither induction with phytohormone applications nor *D. radicum* infestation did affect fresh biomass of heads or shoots (Table 2).

The GLS profile of the broccoli heads harvested 35 or 36 days after planting consisted of two aliphatic GLS, i.e., glucoraphanin and glucoiberin, four indole GLS, i.e., glucobrassicin, neoglucobrassicin and two unknown indole GLS (Table 3). The GLS profiles of the heads were not affected by any of the induction treatments.

**Table 3:** **Mean levels of glucosinolates (μmoles/g dry mass) (±SE) in heads of harvested broccoli**.

**Class/compound**	**Phytohormone treatment**						***D. radicum* infestation**				
	***F***	***df***	***P-value***	**Control**	**JA**	**SA**	***F***	***df***	***P-value***	**Control**	**Infested**
Aliphatic	2.04	2	0.1453	0.40 (0.09)	0.33 (0.09)	0.69 (0.20)	2.44	1	0.1318	0.44 (0.11)	0.78 (0.21)
Glucoraphanin	2.10	2	0.1372	0.33 (0.08)	0.28 (0.08)	0.60 (0.17)	2.24	1	0.1485	0.38 (0.10)	0.67 (0.19)
Glucoiberin	1.27	2	0.2932	0.07 (0.01)	0.05 (0.01)	0.09 (0.03)	3.58	1	0.0712	0.06 (0.01)	0.11 (0.02)
Indole	0.68	2	0.5110	0.87 (0.20)	0.76 (0.26)	1.14 (0.22)	0.55	1	0.4643	0.73 (0.15)	0.93 (0.24)
Glucobrassicin	0.18	2	0.8379	0.13 (0.04)	0.10 (0.04)	0.13 (0.03)	2.72	1	0.1130	0.09 (0.02)	0.16 (0.05)
Neoglucobrassicin	0.38	2	0.6879	0.63 (0.14)	0.58 (0.22)	0.80 (0.17)	0.53	1	0.4722	0.53 (0.12)	0.68 (0.19)
Unknown indole 1	1.64	2	0.2085	0.09 (0.05)	0.06 (0.02)	0.19 (0.07)	0.38	1	0.5440	0.10 (0.03)	0.07 (0.03)
Unknown indole 2	0.14	2	0.8705	0.02 (0.00)	0.02 (0.01)	0.02 (0.00)	0.01	1	0.9048	0.02 (0.00)	0.02 (0.00)
Total	1.25	2	0.2990	1.27 (0.28)	1.09 (0.34)	1.83 (0.38)	1.41	1	0.2472	1.17 (0.26)	1.72 (0.41)

JA, plants treated with jasmonic acid; SA, plants treated with salicylic acid.

## Discussion

Herbivore induced plant responses are known to mediate interactions between insect herbivores that are spatially (i.e., either associated to above- or belowground plant compartments) and temporally separated (Englishloeb et al., 1993). However, the aboveground community-wide consequences of belowground induced responses and their impact on plant productivity have been rarely investigated so far. Here, we demonstrated under realistic field conditions that root induction of broccoli plants by *D. radicum* feeding or phytohormones not only affects the colonization by naturally occurring *D. radicum* females but also the community structure of several leaf herbivore species sharing the same host plant. Our study showed that both leaf-chewing and sap-sucking herbivore abundance can be influenced by root induction. However, the response of herbivores belonging to these feeding guilds were found to differ from one another, depending on the herbivore species, its degree of specialization and the root treatment.

### Effects of root induction treatments on *D. radicum*

The belowground herbivore, *D. radicum* was affected by all root induction treatments. JA application reduced the number of *D. radicum* eggs, while SA did not. This reduction in egg number could be due to a systemic modification of the chemical cues (volatile or not) produced aboveground (van Dam et al., 2004) and used by *D. radicum* to select a host plant (Finch and Collier, 2000). As JA treated plants were slightly smaller than control plants, a visual effect cannot be excluded. Both phytohormone treatments decreased the number of emerging adults. These results suggest that both JA and SA application may modify the suitability of the host plant for the development of the cabbage root-fly larvae. It has previously been shown that JA and SA application on *Brassica* species could induce a local alteration of chemicals such as GLS (Kiddle et al., 1994; van Dam et al., 2004; Pierre et al., 2012) that may affect *D. radicum* larval performance and therefore reduce the number of emerging adults as it was already observed on JA treated broccoli plants (Pierre et al., 2012). Nevertheless, the lower number of emerging adults found on JA treated plants could result not only from a direct effect on larval performance but also from the reduction in the number of eggs laid that has been observed in this study.

Our study showed that *D. radicum* infestation triggered different effects than phytohormone applications on conspecific colonization. The number of eggs laid was strongly increased on root infested plants as previously shown in lab experiments (Baur et al., 1996). Surprisingly the higher number of eggs found on conspecific infested plants did not result in a higher emergence rate of *D. radicum*. This paradox between herbivore preference and performance might result from differential plant induced response between shoots and roots (van Dam and Raaijmakers, 2006). Lower performance on plants harboring a large egg load may also result from a higher density of conspecific competitors on the root as was previously shown on other root herbivores (Robert et al., 2012). In this case, an induced plant that stimulates oviposition may become less suitable for the development of the progeny. In addition, the increase of the larval density could lead to a possible competition for resources between larvae and therefore decrease their survival rate (Ellner et al., 2001; Robert et al., 2012).

For all the induction treatments, the reduction in number of emerging adults may also be due to attraction of natural enemies such as eggs predators (i.e., carabids or staphylinids) (Coaker and Williams, 1963). Previous studies have shown that JA or SA treated plants could be attractive for herbivores natural enemies (van Poecke and Dicke, 2002; Orre et al., 2010). In addition, *D. radicum* infested are attractive for the eggs predators *Aleochara bipustulata* and *Aleochara bilineata* (Ferry et al., 2007).

Finally, in modifying the relative abundance of leaf herbivores, the root induction treatments could also have an indirect effect on the root feeder performances. For instance, it has been shown that the feeding activity of *Brevicoryne brassicae* on *Arabidopsis thaliana* leaves has a negative impact on nematode performances (Kutyniok and Muller, 2012). This emphasizes the fact that sequence of arrival can be an important factor shaping plant mediated interactions between herbivores (Erb et al., 2011b). In this study the root induction treatments may affect the sequence of arrival of several phytophagous species that can consequently impact the colonization processes of other species.

### Effects of root induction treatments on the aboveground community

The aboveground community was more affected by root hormone applications than by previous *D. radicum* root infestation. Nevertheless the effects of root hormone applications on the aboveground herbivore community were somehow surprising. Indeed they did not follow predictable patterns as could be expected based on the general trends reported in the literature, predicting that generalist herbivores tend to avoid induced plants while specialists prefer to colonize them (Martinsen et al., 1998; Kaplan and Denno, 2007; Long et al., 2007; Poelman et al., 2008, 2010). In our study, the generalist foliar herbivores (*My. persicae* and *Ma. brassicae*) were not affected by phytohormone treatments, while the specialists exhibited contrasted effects depending on the herbivore species and the applied hormone. Two specialist foliar herbivores (*B. brassicae* and *Pi. brassicae*) were more abundant on JA treated plants whereas the specialist *P. xylostella* displayed the opposite response. Interestingly, the abundance of three specialist species (*B. brassicae*, *Pi. rapae* and *P. xylostella*) was decreased, but never increased, on SA treated plants. The leaf chewer specialist *P. xylostella* was the only species to respond negatively to both root phytohormone applications, contrasting with experiments performed by Shiojiri et al. (2002); Poelman et al. (2008, 2010), and Mathur et al. (2011) who observed a strong preference of *P. xylostella* for induced *Brassica* plants. However, all these studies focused on aboveground herbivore responses to shoot induction while we worked with root induced plants. Our findings support previous studies showing that shoot and root induction may differentially affect leaf chemistry and herbivore growth (e.g., van Dam and Oomen, 2008; Qiu et al., 2009). For example, (van Dam et al., 2004; van Dam and Oomen, 2008), showed that feral *B. oleracea* plants induced by root treatment with JA displayed higher levels of aliphatic GLS in the leaves, whereas shoot treatment with JA increased indole GLS. Nevertheless, root treatment with SA on *B. oleracea* induced a local but not systemic increase of GLS (van Dam et al., 2004). In our study we observed no change in the final GLS profiles of broccoli heads. However, short term effects of the induction treatments are possible. Indeed, significant effects were observed in feral *B. oleracea* plants at 3–7 days after JA application, and may last for at least 14 days (Jansen et al., 2009). Also, broccoli heads are flower heads and GLS profiles could be dramatically different between flowers and leaves (Smallegange et al., 2007). Alternatively, changes in other plant chemicals such as induced non-GLS secondary metabolites, or above- and belowground reallocation of primary metabolites may contribute to the observed herbivore responses to induced plants (Jansen et al., 2008; van Dam and Oomen, 2008; Poelman et al., 2010; Pierre et al., 2012). For instance, lower sugar levels were found in the leaves of root-induced plants, whereas both sugar and amino acid levels decreased in shoot-induced plants (van Dam and Oomen, 2008). Such plant responses resulted in differential effects on the performance of the generalist *Ma. brassicae* and the specialist *P. rapae* between root- and shoot-induced plants (van Dam and Oomen, 2008). Additional chemical analyses of flowers, leaves and roots at different time windows should be performed to link our results on colonization and performance of the different herbivores surveyed to modification of plant metabolites following induction.

Early season root induction by *D. radicum* larvae only slightly affected the aboveground community structure. The abundance of only one species, *B. brassicae*, was strongly modified on *D. radicum* infested plants. The increased colonization of the specialist aphid *B. brassicae* on root infested plants was already observed on wild mustard, *Sinapis arvensis*, infested with root feeding wireworms, *Agriotes sp*. larvae (Poveda et al., 2005). Previous field studies using a leaf herbivore to induce ozther brassicaceous species also resulted in increased colonization by the sap-sucking leaf specialist *B. brassicae* (Agrawal and Sherriffs, 2001; Poelman et al., 2010).

Taken together, our results show that root inductions by JA and SA have different consequences on the abundance of aboveground herbivores from root infestation by *D. radicum*, suggesting that these phytohormones are not appropriate elicitors to mimic root herbivory. Therefore, our results support growing evidence that other signals may be involved in the induction processes occurring belowground as already suggested by Erb et al. (2012).

### Effects on root induction treatments on the quality of the crop

Our study revealed that SA application did not induce any measurable costs, while JA only induced a diminution of plant heights but other parameters associated to plant quality were not affected. Similar effects on plant growth following JA application have been shown in other crop plants such as radish (*Raphanus sativus* cv. Comet) or maize (*Zea mays* L.) (Ueda and Kato, 1982; Shahzad et al., 2009). Previous infestation with *D. radicum*, on the other hand, resulted in lower visual quality. Considering the average weight of the broccoli heads harvested in our study, all the root inductions carried out resulted in yields sufficient to meet the commercial standards in conventional agriculture (Cerafel Brittany, 2007).

## Conclusion

The present study documented important interactions between belowground induced defences and aboveground herbivore communities. Interestingly, these indirect effects mediated through the plant were found under field conditions despite uncontrolled heterogeneity in biotic and abiotic variables. Furthermore, the induced response effect on herbivore community dynamics followed some communal but also some substantially different patterns compared to the patterns usually observed after leaf induction. These results highlight the need to investigate both the effect of aboveground and belowground induced responses to better understand the mechanisms behind the structuring and functioning of insect herbivore communities in the field.

### Conflict of interest statement

The authors declare that the research was conducted in the absence of any commercial or financial relationships that could be construed as a potential conflict of interest.
